# Effect of Patellar Morphology on the Risk of Osteochondral Fracture after Patellar Dislocation: A Cross‐sectional Study

**DOI:** 10.1111/os.14036

**Published:** 2024-04-01

**Authors:** Zirui Zhou, Qiang Hua, Chenghong Wen, Wenduo Qian, Jide Su, Min Yang, Mingming Lei

**Affiliations:** ^1^ College of Sports Medicine and Health Chengdu Sport University Chengdu China; ^2^ Department of Sports Injury, Affiliated Sports Hospital of Chengdu Sport University Chengdu Sport University Chengdu China

**Keywords:** Computed tomography, Osteochondral fracture, Patellar dislocation, Patellar morphology, Patellofemoral instability

## Abstract

**Objective:**

The risk of osteochondral fracture (OCF) after patellar dislocation has been shown to be related to patellofemoral anatomy, but its relationship to patellar morphology remains unknown. The aim of this study was to investigate the associations between patellar morphology and the risk of OCF after patellar dislocation.

**Methods:**

A total of 140 patients with patellar dislocation between January 2018 and June 2023 were enrolled in this study and divided into two groups. Sixty‐five patellar dislocation patients with OCF were included in the OCF group, while 75 patellar dislocation patients without OCF were included in the non‐OCF group. Computed tomography was used to compare measurements of patellar morphology including Wiberg classification, patellar width and thickness, Wiberg angle, Wiberg index, facet ratio, lateral patellar facet angle, and patellar tilt angle. A logistic regression model was performed to evaluate the correlations between patellar morphology and the risk of OCF after patellar dislocation. Receiver operating characteristic curves were used to calculate the area under the curve (AUC) and determine the diagnostic values of patellar morphology for OCF after patellar dislocation. Subgroup analyses for gender and age were conducted to compare the differences in patellar morphology of PD patients.

**Results:**

Wiberg angle was significantly lower in the OCF group (*p* = 0.017), while Wiberg index (*p* = 0.002) and facet ratio (*p* = 0.023) were significantly higher in the OCF group. According to the results of logistic regression analysis, Wiberg angle (odds ratio [OR] = 0.96, *p* = 0.022) and Wiberg index (OR = 1.105, *p* = 0.032) were the final relevant factors for the occurrence of OCF after patellar dislocation. The AUC was 0.622 (95% confidence interval [CI]: 0.529–0.714) for Wiberg angle, 0.65 (95% CI: 0.558–0.742) for Wiberg index, and 0.702 (95% CI: 0.615–0.788) for the combination of Wiberg angle plus Wiberg index.

**Conclusion:**

Wiberg angle and Wiberg index were independent risk factors for the occurrence of osteochondral fracture after patellar dislocation. Moreover, Wiberg angle, Wiberg index, and the combination of Wiberg angle plus Wiberg index had good predictive diagnostic value for the occurrence of OCF after patellar dislocation.

## Graphical abstract

This study investigated the effect of patellar morphology on the risk of osteochondral fracture after patellar dislocation, and the results showed that Wiberg angle and Wiberg index were associated with the occurrence of osteochondral fracture after patellar dislocation.

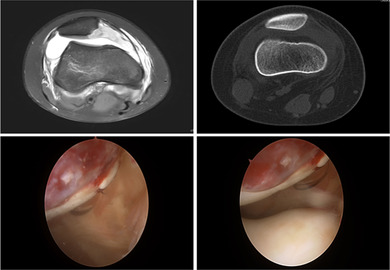

## Introduction

Patellar dislocation (PD) is a common knee disease among pediatric and adolescent patients.^1^ It was reported that the incidence of PD was 147.7 per 100,000 persons.^2^ The risk factors for PD including demographic and path anatomic risk factors have been widely reported.^3^, ^4^ The anatomy of the patella includes the embryo and development of patella, bone anatomy, soft tissue anatomy, and the morphology of the patella.^5^ Askenberger *et al*.^6^ explored the influence of anatomical factors of the patella including the height, tilt, and morphology of the patella on PD. In this study, the morphological factors of the patella were counted as one of the anatomical factors of the patella. Osteochondral fracture (OCF) is a common concomitant injury after PD, which is critical to the choice of treatment for patients with primary PD.^7^ Arthroscopy is the gold standard for identifying OCF, but magnetic resonance imaging (MRI) also has high sensitivity, specificity, and accuracy.^8^, ^9^ Although the risk factors for PD have been widely reported, few studies have explored risk factors for concomitant OCF. Uimonen *et al*.^10^, ^11^ reported on the risk factors and characteristics of OCF caused by PD. They found that the risk of OCF after PD was related to patellofemoral anatomy but they did not evaluate the morphology of the patella.

Whether the morphology of the patella is associated with PD remains controversial. Li *et al*.^12^ found that the morphology of the patella in patients with trochlear dysplasia was different from that in normal individuals. However, Jimenez *et al*.^13^ reported that there was a minimal association between measurements of patellar morphology and trochlear dysplasia. The study populations in the above two studies differed in race, which may have influenced the results. Moreover, Jimenez *et al*.^13^ also raised the question of whether an altered or abnormal patellar shape would preclude groove‐deepening trochleoplasty. No study has explored whether the morphology of the patella influences the choice of surgical modality for PD and leads to differences in postoperative clinical outcomes. The investigation of patellar morphology may provide new insights into the pathology of OCF after PD, which may be beneficial to improve future treatment. In addition, it is not known whether the morphological differences of the patella influence the occurrence of OCFs. Therefore, the current study may be the first to investigate the morphology of the patella in PD patients with OCF.

Therefore, the purpose of the present study was: (i) to determine and compare the difference between the morphology of the patella in PD patients with and without concomitant OCF; and (ii) to identify the independent risk factors and predictive diagnostic factors, including demographic factors and morphologic factors of patella, for the occurrence of OCF after PD.

## Material and Methods

### Patients

The present study was approved by the Ethics Committee of Affiliated Sports Hospital of Chengdu Sport University (August 17th, 2023/No. 008).

**FIGURE 1:**
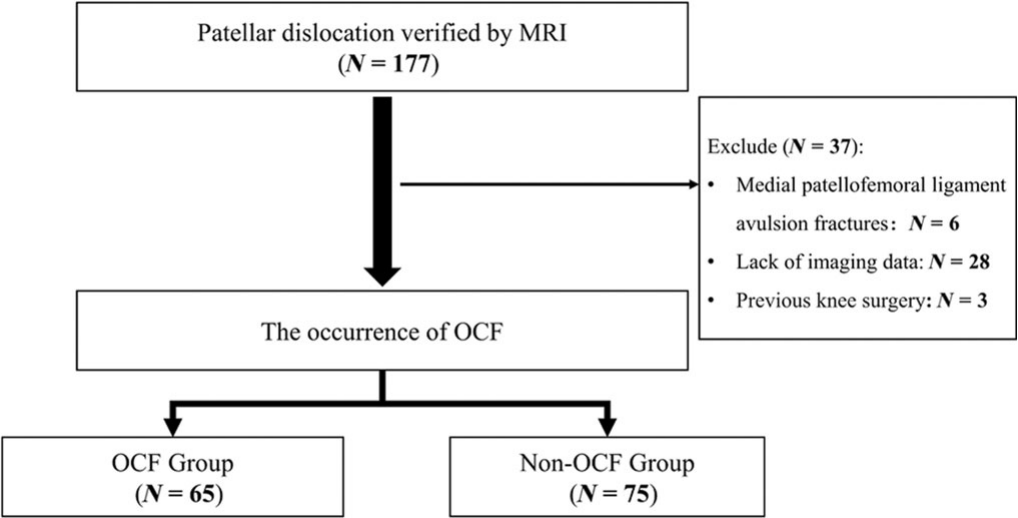
Flow chart of patient selection.

With the approval of the Ethics Committee, the picture archiving and communication systems (PACS) and the electronic database of the hospital were used to retrospectively identify patients treated for PD between January 2018 and June 2023. Each patient was informed before surgery that the relevant imaging data of each patient may be used for scientific research on the premise of not disclosing the personal privacy of the patient because this hospital was a teaching hospital. The inclusion criterion for this study was a diagnosis of PD verified by magnetic resonance imaging (MRI) performed in the current hospital. All MRI examinations were performed on the same 1.5‐T MRI scanner (Multiva, Philips Medical System, Eindhoven, the Netherlands). The presence of OCF was determined by MRI and arthroscopic findings. An osteochondral fragment was defined as an intra‐articular loose body containing components of both cartilage and bone cleaved from the articular surface of the patellofemoral joint. Additionally, preoperative computed tomography (CT) scans were performed to assess the patellar morphology. The exclusion criteria were: (i) previous history of ipsilateral knee surgery; (ii) extra‐articular medial patellofemoral ligament avulsion fractures; and (iii) CT or MRI not performed in the current hospital. Those who had PD with OCF were included in the OCF group, while those without OCF were included in the non‐OCF group (Figure 1). Patient demographics including age, sex, and injury side were retrospectively collected.

### Subgroup Analysis

To investigate the effect of gender and age on the patellar morphology in PD patients, subgroup analysis was conducted based on gender and age. According to gender, OCF group was divided into female OCF group and male OCF group, and non‐OCF group was divided into female non‐OCF group and male non‐OCF group.

According to the grouping criteria of previous study, the subgroup based on age were divided into ≤16 years and >16 years groups.^14^ Consequently, for subgroup analysis for age, OCF group was divided into younger OCF group (≤16 years) and older OCF group (>16 years), and non‐OCF group was divided into younger non‐OCF group (≤16 years) and older non‐OCF group (>16 years).

### Assessment of Patellar Morphology

The assessments of patellar morphology were performed on the axial preoperative CT scans. The current study used the same CT parameters to reduce bias (SOMATOM Sensation 16; Siemens Medical Solutions, Erlangen, Germany). All CT evaluations were performed by two authors (the third and the fourth author) with no knowledge of arthroscopic findings, case history, and preoperative MRI findings. They conducted two measurements with an interval of 2 weeks separately. Several radiologic criteria generally approved in the literature for the assessment of patellar morphology are presented below.

#### Wiberg Classification

**FIGURE 2:**
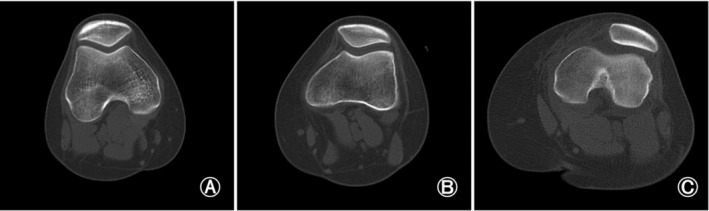
Wiberg classification for patella morphology. (A) Type I: medial facet (concave) equal size as lateral facet (concave). (B) Type II: medial facet (flat, slightly convex) smaller than lateral facet. (C) Type III: medial facet (convex) very much smaller than lateral facet.

Wiberg classification was used to classify the patella form.^15^ Type I: medial facet (concave) equal size as lateral facet (concave) (Figure 2A). Type II: medial facet (flat, slightly convex) smaller than lateral facet (Figure 2B). Type III: medial facet (convex) very much smaller than lateral facet (Figure 2C).

#### Patellar Width

**FIGURE 3:**
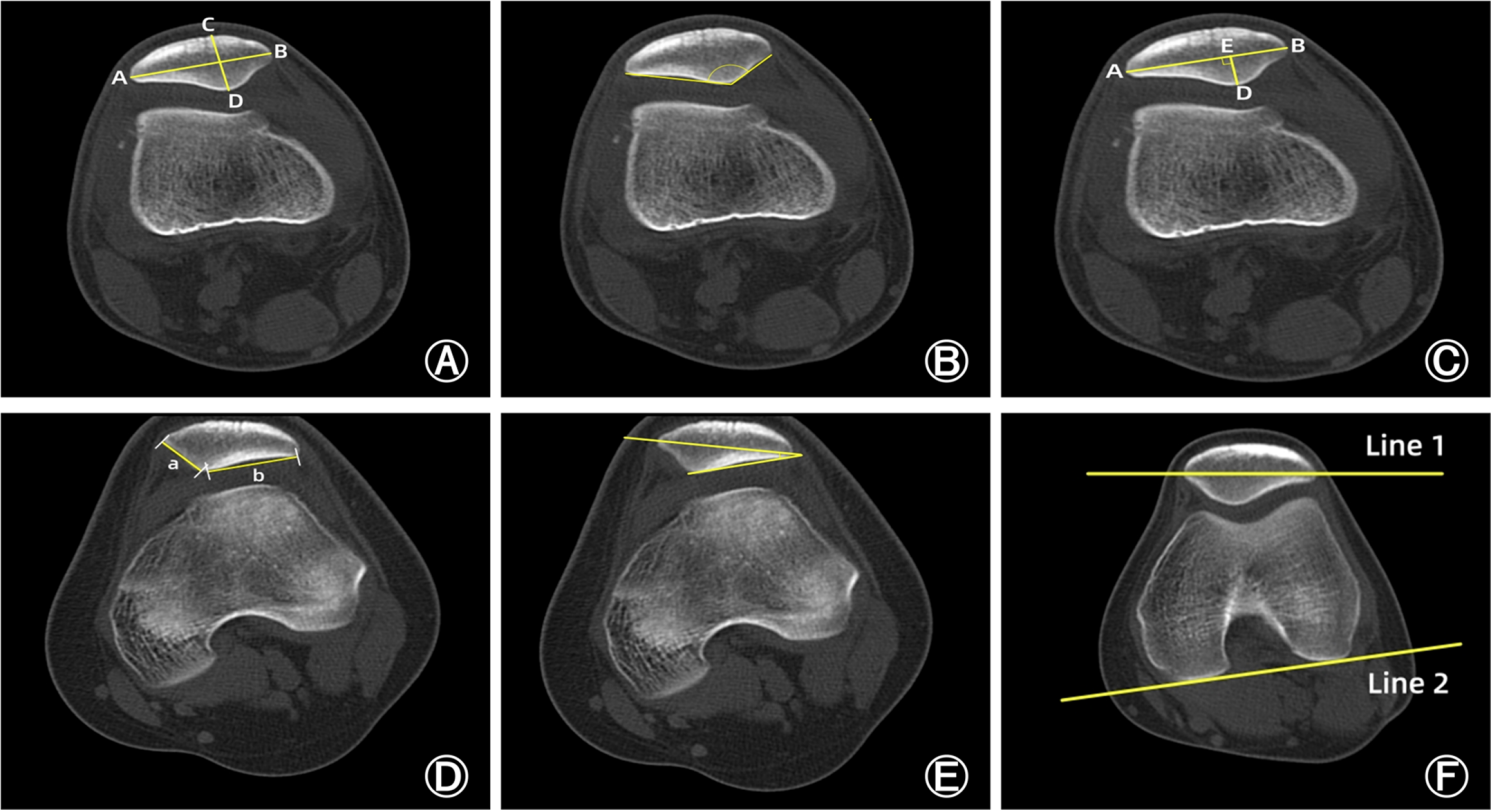
Measurements of the patellar morphology. (A) The patellar width: the length between the lateral (A) and medial edge (B) of the patella in the slide that showed the widest diameter of the patella. The patellar thickness: the length between the patellar front polar (C) and back polar (D). (B) Wiberg angle: the angle between the medial and lateral sides of the patella. (C) Wiberg index: the ratio of the transverse length of the lateral patellar facet (AE) to the patellar width (AB). (D) The facet ratio: the ratio of the length of the lateral side of the patella (a) to the length of the medial side (b). (E) Lateral patellar facet angle: the angle between the patellar transverse axis and the lateral sides of the patella. (F) Patellar tilt angle: the angle between the extension line of the maximum transverse diameter of the patella (line 1) and the tangent to the posterior condyles (line 2).

The patellar width was defined as the length between the lateral (A) and medial edge (B) of the patella in the slide that showed the widest diameter of the patella (Figure 3A). The patellar width reflects the transverse length of the patella.^12^

#### Patellar Thickness

The patellar thickness was defined as the length between the patellar front polar (C) and back polar (D) (Figure 3A). The patellar thickness reflects the vertical length of the patella.^12^

#### Wiberg Angle

Wiberg angle (WA) was defined as the angle between the medial and lateral sides of the patella (Figure 3B). A higher WA represents a flatter articular surface of the patella.^12^, ^15^

#### Wiberg Index

Wiberg index (WI) was defined as the ratio of the transverse length of the lateral patellar facet (AE) to the patellar width (AB) (Figure 3C). A higher WI represents more obvious medial facet dysplasia and lateral facet hyperplasia.^12^, ^15^

#### The Facet Ratio

The facet ratio was defined as the ratio of the length of the lateral side of the patella (a) to the length of the medial side (b) (Figure 3D). An increase of the facet ratio indicated more obvious medial facet dysplasia and lateral facet hyperplasia.^16^

#### Lateral Patellar Facet Angle

Lateral patellar facet angle (LPFA) was defined as the angle between the patellar transverse axis and the lateral sides of the patella (Figure 3E). A sharper LPFA reveals more severe lateral facet hyperplasia.^2^, ^17^

#### Patellar Tilt Angle

Patellar tilt angle (PTA) was defined as the angle between the extension line of the maximum transverse diameter of the patella (line 1) and the tangent to the posterior condyles (line 2) (Figure 3F). An increase of PTA indicated an increase in the inclination of the patella.^2^

### Statistical Analysis

Statistical analysis was performed using SPSS 25.0, with statistical significance set at *p* < 0.05. Continuous data were expressed as mean ± standard deviation (SD) or median with interquartile range (IQR), and categorical data were expressed as absolute numbers or percentages. A 2‐sample *t*‐test was used to compare patellar width, patellar thickness, and WA. Mann–Whitney U test was used to compare the age, WI, facet ratio, LPFA, and PTA. A chi‐square test was used to compare the two groups for gender, the injured side, and Wiberg classification.

A logistic regression model was further performed with the occurrence of OCF as the dependent variable and the patient demographic data and the measurements of patellar morphology with a *p*‐value of < 0.05 as independent variables. In addition, receiver operating characteristic (ROC) curves analysis for patellar morphology was employed to determine the diagnostic values for patellar OCF after PD. The sensitivity and specificity were also calculated using the best cutoff point of patellar morphology with the Youden index for the ROC.

In addition, subgroup analyses for gender and age were conducted. In the subgroup analysis for gender, female OCF group and male OCF group, female non‐OCF group and male non‐OCF group, female OCF group and female non‐OCF group, and male OCF group and male non‐OCF group were compared separately. In the subgroup analysis for age, younger OCF group and older OCF group, younger non‐OCF group and older non‐OCF group, younger OCF group and younger non‐OCF group, and older OCF group and older non‐OCF group were compared separately. Two‐sample *t*‐test and Mann–Whitney U test were used to compare morphological parameters among these groups.

The reliability of imaging measurements was assessed by using the intraclass correlation coefficient (ICC), which quantifies the proportion of the variance of the rating due to variability between measurements. The ICC was interpreted as poor if it was less than 0.4; as marginal, when it was greater than or equal to 0.4 but less than 0.75; and as good, when it was greater than 0.75.

A *post hoc* analysis was also performed to determine whether the sample size of this study had sufficient testing power. The final analysis outcome showed that the sample size of 140 had a sufficient statistical power of 0.83.

## Results

### Patients

Ultimately, A total of 140 patients with PD were evaluated in the current study. Demographic data of the two groups are shown in Table 1. The OCF group included 65 patients with the age of 19 (15.5, 23) years (29 men, 36 women), while the non‐OCF group included 75 patients with the age of 18 (16, 21) years (27 men, 48 women). No significant differences were found in the gender, age, and injured side between two groups.

**TABLE 1:** Demographic data.

	OCF group (*n* = 65)	Non‐OCF group (*n* = 75)	Statistical value	*p*‐value
Age, years, median (IQR)	19 (15.5, 23)	18 (16, 21)	*Z* = 0.44	0.66
Gender, *n* (%)				
Male	29 (44.6%)	27 (36.0%)	*x* ^*2*^ = 1.007	0.299
Female	36 (55.4%)	48 (64.0%)		
Injured side, *n* (%)				
Left	33 (50.8%)	42 (56.0%)	*x* ^*2*^ = 0.383	0.536
Right	32 (49.2%)	33 (44.0%)		

Abbreviations: IQR, interquartile range; OCF, Osteochondral fracture.

### Comparison of Patellar Morphology on CT

Intra‐ and interrater reliabilities of the measurements of patellar morphology are shown in Table 2, indicating that the intraobserver and interobserver reliabilities were high and the method could be used.

**TABLE 2:** Intraobserver and interobserver reliabilities for CT measurement.

	Intraobserver 1	Intraobserver 2	Interobserver
Patellar width	0.988	0.989	0.989
Patellar thickness	0.97	0.958	0.879
WA	0.995	0.995	0.98
WI	0.932	0.966	0.956
Facet ratio	0.973	0.967	0.924
LPFA	0.99	0.99	0.976
PTA	0.992	0.993	0.94

Abbreviations: LPFA, Lateral patellar facet angle; PTA, Patellar tilt angle; WA, Wiberg angle; WI, Wiberg index.

The measurements of patellar morphology were compared between the two groups (Table 3). WA was significantly lower in the OCF group (*p* = 0.017), while WI (*p* = 0.002) and facet ratio (*p* = 0.023) were significantly higher in the OCF group. There were no significant differences in the patella width and thickness, Wiberg classification, LPFA, and PTA between the two groups (*p* < 0.05) (Table 3).

**TABLE 3:** Comparison of measurements.

	OCF group	Non‐OCF group	Statistical value	*p*‐value
Wiberg classification, *n* (%)				
I	1 (1.5%)	3 (4.0%)	*x* ^*2*^ = 4.658	0.092
II	44 (67.7%)	60 (80.0%)		
III	20 (30.8%)	12 (16.0%)		
Patellar width, mm, mean ± SD	40.29 ± 4.09	41.37 ± 3.6	*t* = 1.662	0.099
Patellar thickness, mm, mean ± SD	21 ± 2.32	20.68 ± 2.08	*t* = −0.872	0.385
WA, degrees, mean ± SD	120.25 ± 10.93	124.6 ± 10.34	*t* = 2.418	**0.017**
WI, median (IQR)	0.64 (0.61, 0.68)	0.61 (0.59, 0.65)	*Z* = −3.058	**0.002**
Facet ratio, median (IQR)	1.69 (1.5, 1.95)	1.55 (1.41, 1.77)	*Z* = −2.267	**0.023**
LPFA, degrees, median (IQR)	20 (17.45, 23.3)	19.7 (17.1, 22.8)	*Z* = −0.545	0.586
PTA, degrees, median (IQR)	16.2 (11.75, 23.15)	16.4 (10.8, 22.3)	*Z* = −0.698	0.485

*Note*: Significant differences are indicated in bold.

Abbreviations: IQR, interquartile range; LPFA, Lateral patellar facet angle; OCF, osteochondral fracture; PTA, Patellar tilt angle; SD, standard deviation; WA, Wiberg angle; WI, Wiberg index.

### Correlation Analysis of Patellar Morphology with OCF after PD

Gender, age, WA, WI, and facet ratio were then included in a multivariate logistic regression analysis. The multivariate logistic regression analysis showed WA (odds ratio [OR] = 0.96, *p* = 0.022) and WI (OR = 1.105, *p* = 0.032) were the final relevant factors for the occurrence of OCF after PD (Table 4).

**TABLE 4:** Multivariate logistic regression analysis of age, gender, WA, WI, and facet ratio for the occurrence of OCF after PD.

	OR	95% CI	*p*‐value
Age	1.007	0.995–1.062	0.802
Gender	1.250	0.597–2.616	0.555
WA	0.960	0.927–0.994	**0.022**
WI	1.105	1.009–1.211	**0.032**
Facet ratio	0.575	0.122–2.714	0.485

*Note*: Significant differences are indicated in bold.

Abbreviations: CI, confidence interval; OR, odds ratio; WA, Wiberg angle; WI, Wiberg index.

### Diagnostic Performance of Patellar Morphology

**FIGURE 4:**
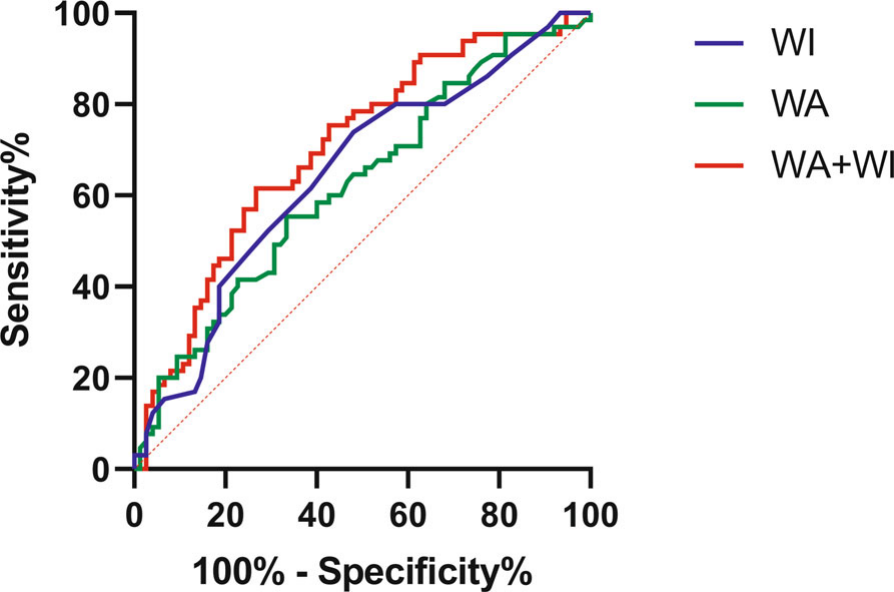
Receiver operating characteristic (ROC) curve analysis for Wiberg angle (WA), Wiberg index (WI), and combination of WA plus WI. ROC analysis showed that the area under the curve (AUC) was 0.622 (95% CI: 0.529–0.714) for WA, 0.65 (95% CI: 0.558–0.742) for WI, and 0.702 (95% CI: 0.615–0.788) for the combination of WA plus WI.

ROC curve analysis was further performed to explore the diagnostic values of WA and WI for OCF after PD. As shown in Table 5 and Figure 4, the AUC was 0.622 (95% confidence interval [CI]: 0.529–0.714) for WA, 0.65 (95% CI: 0.558–0.742) for WI, and 0.702 (95% CI: 0.615–0.788) for the combination of WA plus WI. The cutoff values calculated by Youden index (sensitivity + specificity −1) were 121.6, 0.62, and 0.47, respectively (Table 5).

**TABLE 5:** ROC analysis of diagnostic performance characteristics of WA and WI.

	AUC	95% CI	Cutoff value	Sensitivity (%)	Specificity (%)
WA	0.622	0.529–0.714	121.6	55.4	66.7
WI	0.65	0.558–0.742	0.62	72.3	57.3
WA + WI	0.702	0.615–0.788	0.47	61.5	73.3

Abbreviations: AUC, Area under curve; CI, confidence interval; ROC, Receiver operating characteristic; WA, Wiberg angle; WI, Wiberg index.

### Subgroup Analysis for Gender

Table 6 shows the results of subgroup analysis for gender. In the OCF group, there were 36 female and 29 male patients, while in the non‐OCF group, there were 48 female and 27 male patients. In both the OCF and the non‐OCF group, male patients had wider and thicker patella than female patients (*p* < 0.001, Table 6). Moreover, male patients in the non‐OCF group had higher WI (*p* = 0.006) and facet ratio (*p* = 0.005), and lower LPFA (*p* = 0.021) than female patients in the non‐OCF group (Table 6). Further comparison between male and female subgroups in the OCF and non‐OCF groups showed that PD patients with OCF had thinner patellar than PD patients without OCF in both male (*p* = 0.042) and female subgroup (*p* = 0.038, Table 6). In addition, PD patients with OCF had lower WA (*p* = 0.003), higher WI (*p* < 0.001) and facet ratio (*p* = 0.014) than PD patients without OCF in the female subgroup (Table 6).

**TABLE 6:** Subgroup analysis results for gender.

	OCF group			Non‐OCF group				
	Female (*n* = 36)	Male (*n* = 29)	*p*‐value	Female (*n* = 48)	Male (*n* = 27)	*p*‐value	*p*‐value (female)	*p‐*value (male)
Age, years	19 (15–22)	18 (16–26)	*p* = 0.255	18 (16–20)	17 (16–21)	*p* = 0.0996	*p* = 0.982	*p* = 0.454
Injured side								
Left	18 (50.0%)	15 (51.7%)	*p* = 0.890	25 (52.1%)	17 (63.0%)	*p* = 0.362	*p* = 0.850	*p* = 0.826
Right	18 (50.0%)	14 (48.3%)		23 (47.9%)	10 (37.0%)			
Wiberg classification, *n* (%)								
I	1 (2.8%)	0 (0.0%)	*p* = 0.778	3 (6.3%)	0 (0.0%)	*p* = 0.145	*p* = 0.068	*p* = 0.643
II	23 (63.9%)	21 (72.4%)		39 (81.3%)	21 (77.8%)			
III	12 (33.3%)	8 (27.6%)		6 (12.5%)	6 (22.2%)			
Patellar width, mm	38.22 ± 2.85	42.86 ± 3.96	***p* < 0.001**	39.49 ± 2.66	44.71 ± 2.46	***p* < 0.001**	***p* = 0.038**	***p* = 0.042**
Patellar thickness, mm	20.07 ± 1.99	22.16 ± 2.19	***p* < 0.001**	19.79 ± 1.67	22.27 ± 1.79	***p* < 0.001**	*p* = 0.476	*p* = 0.841
WA, degrees	118.58 ± 10.63	122.31 ± 11.12	*p* = 0.173	125.42 ± 9.63	123.13 ± 11.54	*p* = 0.362	***p* = 0.003**	*p* = 0.788
WI	0.65 ± 0.06	0.66 ± 0.08	*p* = 0.448	0.61 ± 0.04	0.65 ± 0.07	***p* = 0.006**	***p* < 0.001**	*p* = 0.599
Facet ratio	1.71 ± 0.36	1.77 ± 0.31	*p* = 0.426	1.53 ± 0.23	1.81 ± 0.44	***p* = 0.005**	***p* = 0.014**	*p* = 0.754
LPFA, degrees	19.85 (17.45–25.15)	20 (17.35–21.9)	*p* = 0.621	20.6 (17.33–23.95)	19 (16.5–20.1)	***p* = 0.021**	*p* = 0.935	*p* = 0.113
PTA, degrees	16.65 (11.73–23.23)	15.9 (12.75–22.1)	*p* = 0.900	14.9 (10.4–22.2)	16.8 (13.1–24)	*p* = 0.292	*p* = 0.236	*p* = 0.712

*Note*: Significant differences are indicated in bold.

Abbreviation: LPFA, Lateral patellar facet angle, OCF, osteochondral fracture; PTA, Patellar tilt angle; WA, Wiberg angle; WI, Wiberg index.

### Subgroup Analysis for Age

Table 7 shows the results of subgroup analysis for age. In the OCF group, there were 23 younger and 42 older patients, while in the non‐OCF group, there were 23 younger and 52 older patients. Younger patients had lower WA (*p* = 0.020) in the OCF group, and higher WI (*p* = 0.027) and facet ratio (*p* = 0.008) in the non‐OCF group (Table 7). Further comparison between younger and older subgroups in the OCF and non‐OCF groups showed that PD patients with OCF had lower WA than PD patients without OCF in the younger subgroup (*p* < 0.001), while in the older group, PD patients with OCF had higher WI (*p =* 0.004) and facet ratio (*p =* 0.017) than PD patients without OCF (Table 7).

**TABLE 7:** Subgroup analysis results for age.

	OCF group			Non‐OCF group				
	≤ 16 years (*n* = 23)	>16 years (*n* = 42)	*p‐*value	≤ 16 years (*n* = 23)	>16 years (*n* = 52)	*p*‐value	*p‐*value (≤ 16 years)	*p*‐value (>16 years)
Gender								
Male	9 (39.1%)	20 (47.6%)	*p* = 0.510	7 (30.4%)	20 (38.5%)	*p* = 0.504	*p* = 0.536	*p* = 0.372
Female	14 (60.9%)	22 (52.4%)		16 (69.6%)	32 (61.5%)			
Injured side								
Left	11 (47.8%)	22 (52.4%)	*p* = 0.725	13 (56.5%)	29 (55.8%)	*p* = 0.952	*p* = 0.555	*p* = 0.743
Right	12 (52.2%)	20 (47.6%)		10 (43.5%)	23 (44.2%)			
Wiberg classification, *n* (%)								
I	0 (0.0%)	1 (2.4%)	*p* = 0.613	1 (4.3%)	2 (3.8%)	*p* = 0.969	*p* = 0.189	*p* = 0.476
II	14 (60.9%)	30 (71.4%)		18 (78.3%)	42 (80.8%)			
III	9 (39.1%)	11 (26.2%)		4 (17.4%)	8 (15.4%)			
Patellar width, mm	39.52 ± 3.72	40.71 ± 4.26	*p* = 0.263	40.91 ± 3.64	41.58 ± 3.61	*p* = 0.461	*p* = 0.208	*p* = 0.290
Patellar thickness, mm	20.68 ± 2.01	21.18 ± 2.48	*p* = 0.414	20.19 ± 2.07	20.89 ± 2.07	*p* = 0.179	*p* = 0.418	*p* = 0.546
WA, degrees	116.02 ± 9.84	122.56 ± 10.91	***p* = 0.020**	125.41 ± 8.85	124.24 ± 10.99	*p* = 0.656	***p* < 0.001**	*p* = 0.462
WI	0.65 (0.62–0.68)	0.63 (0.61–0.68)	*p* = 0.484	0.63 (0.59–0.67)	0.61 (0.57–0.64)	***p* = 0.027**	*p* = 0.215	***p* = 0.004**
Facet ratio	1.7 (1.56–1.99)	1.68 (1.48–1.92)	*p* = 0.459	1.66 (1.55–1.99)	1.49 (1.34–1.72)	***p* = 0.008**	*p* = 0.817	***p* = 0.017**
LPFA, degrees	20 (17.6–24.7)	19.75 (13.38–22.43)	*p* = 0.721	18.8 (17–22)	20.1 (17.2–23.55)	*p* = 0.275	*p* = 0.277	*p* = 0.918
PTA, degrees	14.7 (11.2–23.3)	16.5 (13.38–23.13)	*p* = 0.243	17.4 (10.4–21.9)	15.4 (10.85–24.23)	*p* = 0.913	*p* = 0.869	*p* = 0.280

*Note*: Significant differences are indicated in bold.

Abbreviations: LPFA, Lateral patellar facet angle; PTA, Patellar tilt angle; OCF, osteochondral fracture; WA, Wiberg angle, WI, Wiberg index.

### Imaging and Arthroscopic Findings

**FIGURE 5:**
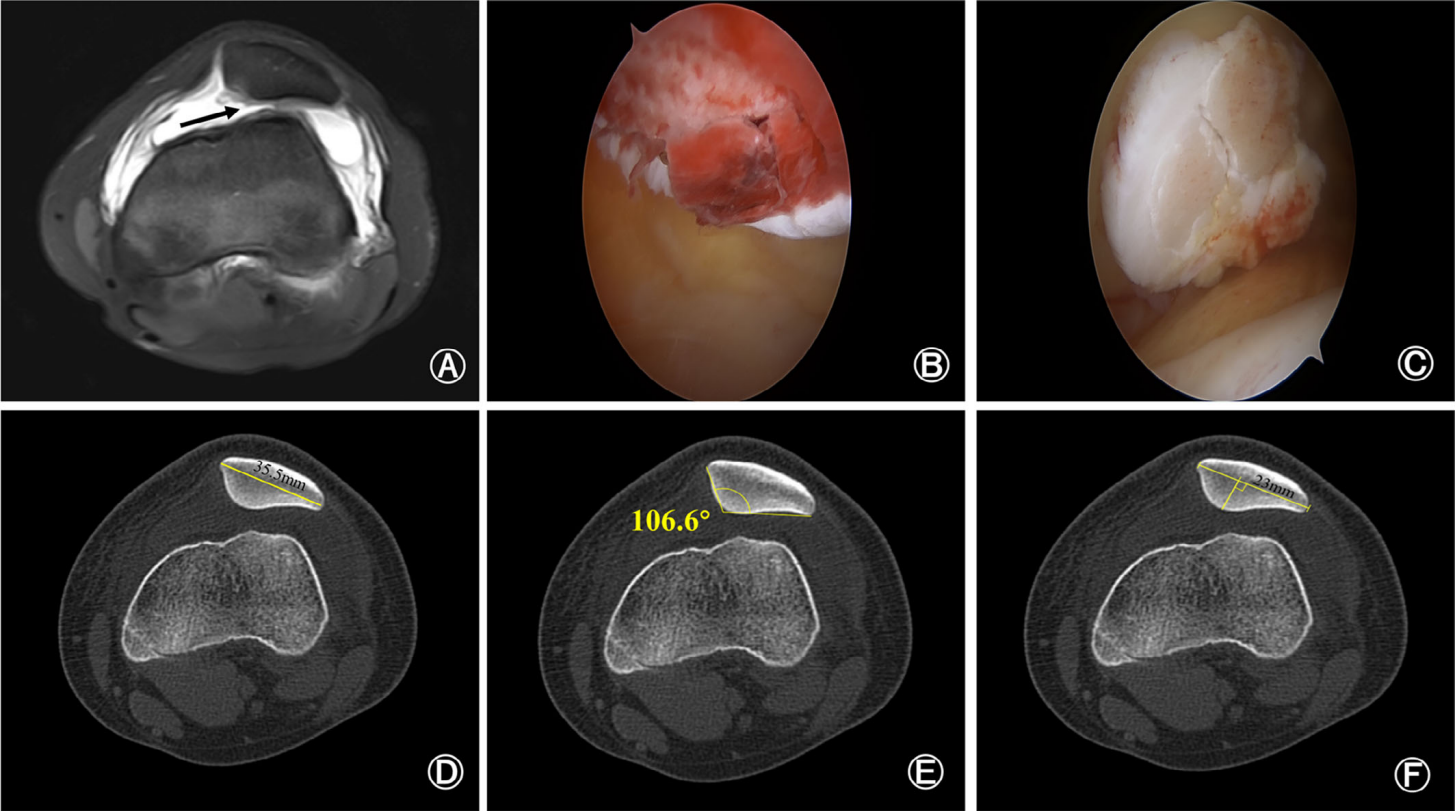
Preoperative magnetic resonance imaging and intraoperative arthroscopic findings of a 14‐year‐old patellar dislocation patient with osteochondral fracture. (A) Preoperative magnetic resonance imaging showed the fracture site on patella (black arrow). (B, C) The fracture site (B) and the fracture block covering the cartilage (C) was found under arthroscopy. (D–F) Preoperative computed tomography showed that the width of patella was 35.5 millimeters (D), Wiberg angle was 106.6 degrees (E) and Wiberg index was 0.65 (F).

**FIGURE 6:**
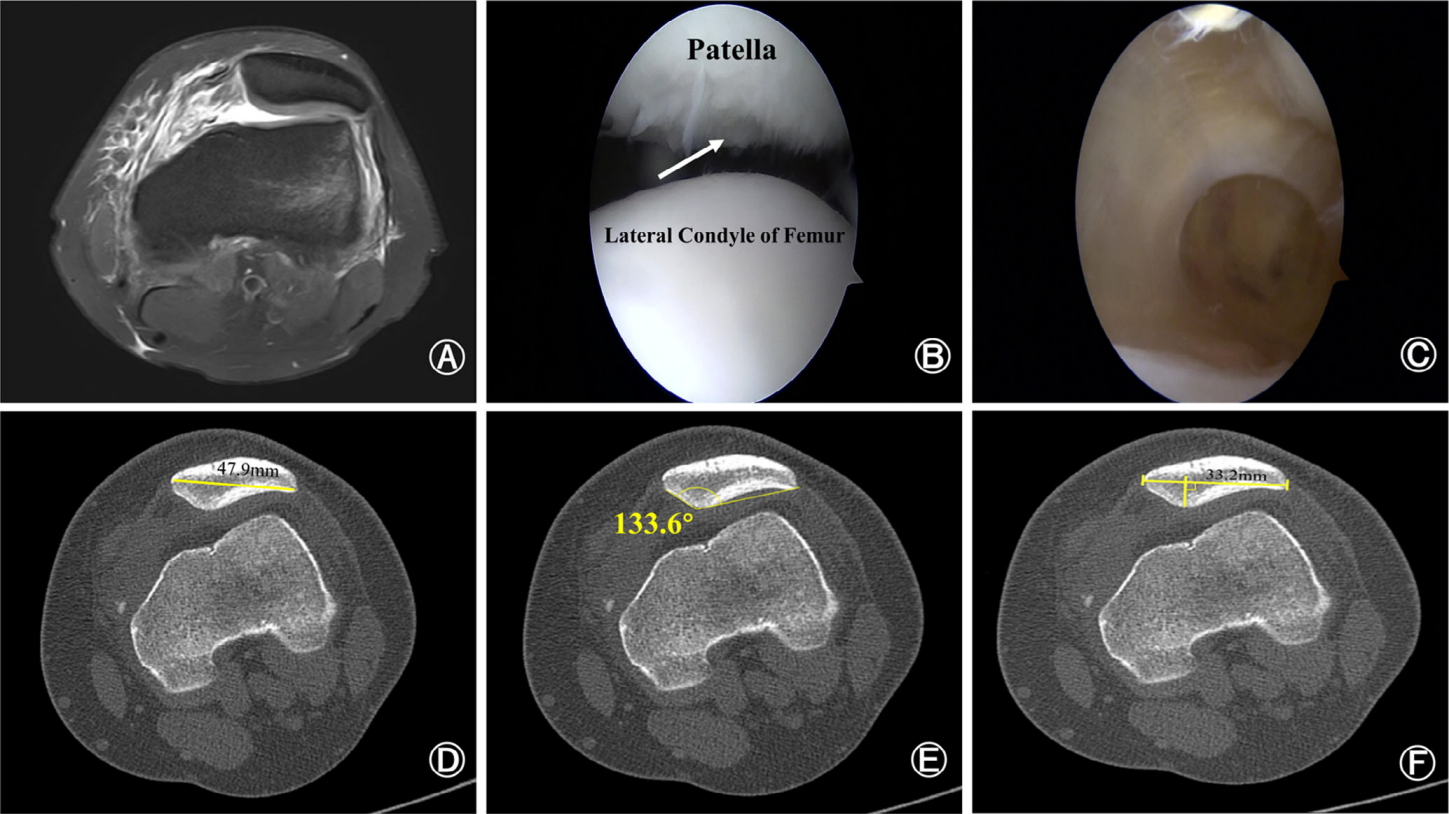
Preoperative magnetic resonance imaging and intraoperative arthroscopic findings of a 21‐year‐old patellar dislocation patient without osteochondral fracture. (A) Preoperative magnetic resonance imaging showed that the medial patellofemoral ligament was injured. (B) The injury site on patella but no osteochondral fracture (white arrow). (C) There were no intra‐articular loose body. (D–F) Preoperative computed tomography showed that the width of patella was 47.9 millimeters (D), Wiberg angle was 133.6 degrees (E) and Wiberg index was 0.7 (F).

Preoperative imaging as well as intraoperative arthroscopic findings were used to determine the presence of OCF, which determined grouping. Patients in the OCF group had OCF on preoperative MRI, or the fracture area and the fracture block covering the cartilage were found under arthroscopy (Figure 5), while all patients in the non‐OCF group had no osteochondral fracture on preoperative MRI and intraoperative arthroscopic findings (Figure 6).

## Discussion

The main finding of this study was that PD patients with OCF have lower WA and higher WI compared to PD patients without OCF, which may contribute to the increased risk of OCF during PD. Additionally, both WA and WI had predictive diagnostic values for the occurrence of OCF after PD, and the combination of WA plus WI had better predictive diagnostic values. To the best of our knowledge, the present study for the first time investigated the patellar morphology of PD patients with OCF.

### Previous Studies on the Morphology of the Patella in PD Patients

Femoral trochlea dysplasia is now widely recognized as one of the major pathogenic factors of PD and has been shown to be the most accurate predictor of recurrent patellar dislocation, mainly because the femoral trochlea provides a sliding track for the patella.^6^, ^18^, ^19^ Li *et al*.^12^ found that the patellar morphology of individuals with femoral trochlear dysplasia was significantly different from that of individuals with normal femoral trochlear morphology. Their research showed that the patella in trochlear dysplasia had a smaller width, a thinner thickness, a lengthened lateral facet, and a more flattened articular facet.^12^ Moreover, femoral trochlear dysplasia has been implicated in the occurrence of OCF following PD in several previous studies although knowledge of the risk factors associated with OCF after PD remains scarce.^10^, ^20^ Uimonen *et al*.^10^ found that the risk of OCF after PD was related to patellofemoral anatomy. However, there are relatively few articles studying the morphology of the patella in PD patients with OCF.

### Possible Mechanisms of Abnormal Patellar Morphology Leading to Following OCFs

In the present study, PD patients with OCF had lower WA and higher WI compared to PD patients without OCF. A lower WA represents a decrease in the contact area between the patellar articular surface and the femoral trochlea articular surface, while a higher WI represents more obvious medial facet dysplasia and lateral facet hyperplasia.^2^, ^12^ Van Haver *et al*.^21^ found that an extroverted and outward‐moving patella resulted in increased pressure on the articular surface of the patella and a significant decrease in the area in contact with the femoral trochlea. The present study showed that the articular surface of the patella in PD patients with OCF was more convex, which resulted in greater pressure on the articular surface of the patella. According to the basic principles of physics, the smaller the contact surface, the greater the pressure exerted on the surface. Uimonen *et al*.^10^ suggested that patients with patellar dislocation with a smaller articular surface of the patella may have a higher risk of OCF, which is consistent with our study. Previous studies have shown that cyclic loading on the joint surface activating endochondral ossification and degradation of subchondral bone tissue matrix may lead to the formation of local micro cracks in subchondral bone, which was thought to be a key factor in reducing the fracture resistance of the whole bones.^22^, ^23^ Therefore, the more convex articular surface of the patella bears greater stress, which may be one of the key reasons for the occurrence of osteochondral injury after patellar dislocation.

Uimonen *et al*.^10^ found that the femoral trochlear facet joint asymmetry was more severe in PD patients with patellar OCF. During growth, the trochlea develops alongside the patella, and the articular surfaces conform to each other.^10^, ^23^ Therefore, they suggested that dysplasia of the articular surface of the patella was associated with an increased risk of patellar OCF. However, their suspicion was not confirmed because their study did not evaluate the patellar morphology of PD patients. The current study found that PD patients with OCF had higher WI, which suggested that dysplasia of the patellar articular surface may indeed be associated with an increased risk of patellar OCF. Several previous studies have reported a shorter length of the medial articular surface of the patella in PD patients, which is more consistent with the Wiberg type III patella.^15^, ^24^, ^25^ Unfortunately, the current study did not find an association between Wiberg type III patella and patellar OCF. Although Askenberger *et al*.^6^ and Servien *et al*.^26^ found that Wiberg type III patella were more common in PD patients, their study did not include Asians. In the current study, Wiberg type II patella were more common. Wiberg type III patella may be relatively rare in this study so no significant association was found between Wiberg type III patella and OCF after PD. Therefore, we believe that the type of patella may be different in different races, but this still needs to be confirmed by further studies.

### Gender‐related Differences in Patellar Morphology of PD Patients

Yi *et al*. found that male sex was a risk factor for osteochondral injuries after PD.^14^, ^27^ Retzky *et al*.^28^ also found that male sex was an independent risk factor for OCF after PD in pediatric patients. Although this study did not confirm the above conclusions, subgroup analysis for gender was conducted in this study to explore whether differences in patellar morphology led to the differences in the incidence of OCF between patients of different genders. Subgroup analysis for gender in the present study found that PD patients with OCF had narrower patella than PD patients without OCF in both male and female subgroups. However, Table 3 in this study showed that there was no statistical difference in patellar width between PD patients with OCF and PD patients without OCF. This may be due to differences in patellar width between men and women. Gender may be a confounding factor between patellar width and the occurrence of OCF after PD. Subgroup analysis for gender also found that patellar width was significantly higher in men than in women in both the OCF group and the non‐OCF group, consistent with previous study.^12^ A large amount of studies have found that abnormal patellofemoral anatomy is more common in women, resulting in a significantly higher risk of patellar dislocation in women than in men.^1^, ^29^, ^30^ Li *et al*.^12^ held the opinion that a larger Q Angle in women would produce a larger pull force on the patella. To reduce the pull force and make the patellofemoral joint more stable, the articular surface of the patella in women would develop more prominently. This study also found that PD female patients with OCF had lower WA, higher WI, and higher facet ratio than PD female patients without OCF. A Lower WA, and a higher WI and facet ratio represent the dysplasia of the articular surface of the patella.^2^, ^16^ In the two male subgroups, there were no significant differences in patellar morphological parameters except patellar width. This suggests that dysplasia of the articular surface of the patella may lead to OCF after PD in female patients. In addition, there were no statistical differences in WA, WI, the facet ratio, LPFA, and PTA between female patients and male patients in the OCF group. Differences in the incidence of OCF after PD between patients of different genders may not be attributed to differences in patellar morphology between genders.

### Age‐related Differences in Patellar Morphology of PD Patients

In the subgroup analysis for age of this study, the dysplasia of articular surface of patella was more common in younger patients (≤ 16 years). Ossification of the patella begins at age 3 and age 5 in girls and boys, respectively, and is complete by age 13–16 and by age 15–18 in girls and boys, respectively.^31^ Therefore, the authors held the view that younger patients in this study were skeletally immature, which may lead to the dysplasia of the articular surface of the patella. Bone maturity is not only a risk factor for recurrent patellar dislocation, but it also increases the risk of OCF after PD.^27^, ^32^, ^33^ Flachsmann *et al*.^34^ found that uncalcified cartilage in skeletally immature patients weaves with subchondral bone, which may lead to the adolescent joint particularly susceptible to osteochondral shear fracture. The subgroup analysis for age also found that the articular surface of the patella was more common in PD patients with OCF, regardless of age. However, due to the study design, this study aimed to investigate the associations between patellar morphology and the risk of OCF after PD. The present study controlled the baseline data to reduce the bias. Although this study did not confirm gender and age as risk factors for OCF after PD, differences in patellar morphology were found between genders and ages of PD patients. Future studies could further explore whether gender and age influence patellar morphology in PD patients and their relationship to OCF after PD.

### Strengths and Limitations

This study has several strengths. The main strength of this study lies in the comprehensive exploration of patellar morphology in PD patients with OCF. The authors believed that the current study may be the first to investigate the patellar morphology in PD patients with OCF. Additionally, this study also tested the conjecture raised in a previous study. Although Uimonen *et al*.^10^ speculated that dysplasia of the articular surface of the patella was associated with an increased risk of OCF after PD, their study did not investigate the patellar morphology in PD patients with OCF. Interestingly, the current study confirmed that the dysplasia of the articular surface of the patella was more pronounced in PD patients without OCF than in PD patients with OCF. Finally, the findings of this study would be beneficial for the predictive diagnosis of PD patients as well as for preoperative planning.

However, there were several limitations in the current study. First, this study included only patients who had undergone surgical treatment for PD. Some patients not treated by operation were excluded from this study because of a lack of imaging data or loss of follow‐up, which may result in a smaller study sample size. Second, the study was retrospective, which led to missing data on some potential causative factors, such as the mechanism of injury. Third, the grade of patellar cartilage damage was not recorded in the non‐OCF group in this study. The question of whether patellar morphology affects the grade of patellar cartilage damage in PD patients without OCF is unknown. Finally, the study was a single‐center study, which cannot be generalized to all populations.

## Conclusion

Wiberg angle and Wiberg index were independent risk factors for the occurrence of osteochondral fracture after patellar dislocation. Moreover, Wiberg angle, Wiberg index, and the combination of Wiberg angle plus Wiberg index had good predictive diagnostic value for the occurrence of osteochondral fracture after patellar dislocation.

## Funding Information

This study was granted by Sports Medicine Key Laboratory of Sichuan Province (2023‐A056).

## Conflict of Interest Statement

The authors declare that they have no competing interests.

## Ethical Statement

This study was performed in line with the principles of the Declaration of Helsinki. Ethical approval was granted by the Ethics Committee of Affiliated Sports Hospital of Chengdu Sport University (August 17, 2023/No. 008).

## Author Contributions

All authors had full access to the data in the study and take responsibility for the integrity of the data. Zirui Zhou: the conception and design of study, drafting the article, and revising it critically for important intellectual content. Qiang Hua: drafting the article and manuscript review. Chenghong Wen, Wenduo Qian, and Jide Su: acquisition of data; analysis and interpretation of data. Min Yang: analysis and interpretation of data and drafting the tables and figures. Mingming Lei: the conception and design of study, revising it critically for important intellectual content, and final approval of the version to be submitted.

## Data Availability

The datasets used or analyzed during the current study are available from the corresponding author on reasonable request.
